# Aducanumab Therapy to Treat Alzheimer's Disease: A Narrative Review

**DOI:** 10.1155/2022/9343514

**Published:** 2022-03-09

**Authors:** Semira Abdi Beshir, A. M. Aadithsoorya, Affana Parveen, Sheron Sir Loon Goh, Nadia Hussain, Vineetha Bharathan Menon

**Affiliations:** ^1^Clinical Pharmacy & Pharmacotherapeutics Department, Dubai Pharmacy College, Dubai, UAE; ^2^College of Medicine, Gulf Medical University, Ajman, UAE; ^3^College of Pharmacy, Gulf Medical University, Ajman, UAE; ^4^Department of Primary Care Medicine, Faculty of Medicine, University of Malaya, Kuala Lumpur, Malaysia; ^5^Department of Pharmaceutical Sciences, College of Pharmacy, Al Ain University, Al Ain, Abu Dhabi, UAE; ^6^Department of Pharmacy Practice, College of Pharmacy, Gulf Medical University, Ajman, UAE

## Abstract

**Background:**

Aducanumab, a new monoclonal antibody that targets *β*-amyloid aggregates, has been granted conditional approval by the U.S. FDA for treatment of mild Alzheimer's disease (AD). The approval of this drug without a confirmed significant clinical impact has resulted in several debates.

**Objective:**

In this narrative review, aducanumab approval-related controversy, the drug's pharmacokinetics and pharmacodynamic characteristics, evidence from the efficacy and safety trials of aducanumab, implications of the drug approval, and the future directions in the management of patients with AD are summarized.

**Methods:**

Using relevant keywords, Google Scholar, Web of Science, and MEDLINE databases and manufacturer's website were searched.

**Results:**

Infusion of aducanumab at a higher dose resulted in a modest slowing of cognitive decline among patients with mild cognitive impairment or early-onset AD dementia. The drug however can cause amyloid-related imaging abnormalities. Due to modest impact on cognition, the use of this drug by patients with AD will most likely be limited. The manufacturer is required to run an extended phase IIIb trial to verify the benefit of this drug. Access to therapy requires a careful selection of patients and periodic monitoring to ensure the optimal use of the drug.

**Conclusion:**

Despite the limitations, aducanumab is the first disease-modifying therapy approved for the treatment of AD. Aducanumab addresses a part of the pathogenesis of AD; therefore, drugs that can act on multiple targets are needed. In addition, the search for preventive strategies, validated plasma-based assays, and newer drugs for AD, which are effective, safe, convenient, and affordable, is vital.

## 1. Introduction

Alzheimer's disease (AD) is one of the most prevalent neurodegenerative diseases resulting in progressive cognitive decline. Over 55 million individuals worldwide are affected by this debilitating disease which has a detrimental impact on quality of life, productivity, and the economy [1]. Due to the increased aging population, the prevalence of Alzheimer's disease is anticipated to increase [1, 2]. The causes of AD remain unclear; however, the occurrence of AD is associated with aging, environmental, genetic, and lifestyle factors. Aggregates of *β*-amyloid protein peptides and neurofibrillary tangles (NFT) which damage the neurons are thought to be linked with the pathogenesis of AD [3]. Neuronal loss, gliosis, genetic mutations, cerebrovascular amyloidosis, and reduced concentrations of the neurotransmitter are also cited as additional factors [3, 4].

**Figure 1:**
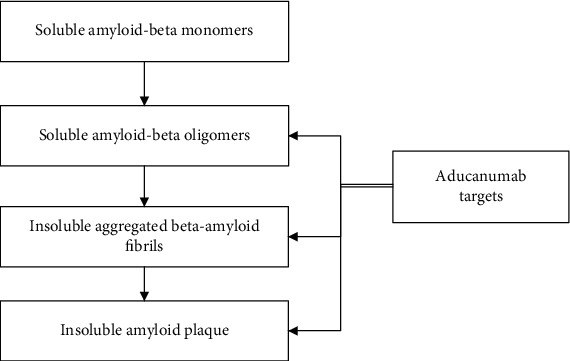
Schematic representation of aducanumab targets (soluble and insoluble beta-amyloid peptides); source [73].

The cure for AD remains elusive, despite the advances made in AD drug research and development. Most drugs that are developed for AD are aimed at reducing the symptoms associated with the disease rather than targeting the underlying cause of the disease [5]. The common drugs used for the symptomatic management of patients with Alzheimer's include cholinesterase inhibitors (donepezil, rivastigmine, and galantamine), neuroprotective N-methyl-D-aspartate (NMDA) receptor antagonist (memantine) [6, 7], or combination therapy. Aducanumab was first manufactured by Neurimmune, and later in 2007, the license was sold to Biogen [8]. On June 7, 2021, FDA granted the conditional accelerated approval for aducanumab as the first disease-modifying agent for treating AD. This monoclonal antibody targets soluble (*β*-amyloid oligomers) and insoluble aggregates of *β*-amyloid proteins (fibrils and *β*-amyloid plaques) as depicted in Figure 1 [9].

Despite the data from several studies indicating that *β*-amyloid is linked to AD, most trials of anti-amyloid drugs have failed to show a significant impact on cognition [10, 11]. Aducanumab when used at higher doses is shown to have a modest impact on the cognitive decline of patients at the early stage of Alzheimer's dementia or mild cognitive impairment (MCI) [12–14]. However, aducanumab does not reverse prior memory loss that is associated with AD. The conditional approval was granted contingent on the conduct of further studies although the majority of U.S. FDA advisory board members voted against the drug's approval.

Though most patient advocates are pleased with the hope of having a new drug to treat AD, the approval of aducanumab creates unnecessary uncertainties for patients, clinicians, and researchers. The drug is under review in Japan, Brazil, and Australia [15]. The European Medicines Agency has recently voted against the approval of aducanumab for use in European countries. In this review, evidence from clinical trials of aducanumab, implications of aducanumab approval, and future directions in the management of patients with Alzheimer's disease are discussed.

## 2. Materials and Method

The literature search was conducted in August 2021. Relevant search terms such as aducanumab OR anti beta-amyloid therapy OR immunotherapy OR monoclonal antibody and Alzheimer's disease OR dementia OR mild cognitive impairment were used. Google Scholar, Web of Science, and MEDLINE databases and manufacturer's website were searched to identify clinical trials, reviews, and updates on aducanumab in patients with AD.

## 3. Pharmacokinetics and Pharmacodynamic Characteristics of Aducanumab

According to the “amyloid theory,” aggregates of amyloid-*β* oligopeptides are linked with Alzheimer's disease [16]. Aducanumab is a fully human IgG1 monoclonal antibody with a high affinity that acts by breaking down these *β*-amyloid aggregates into smaller oligopeptides or amino acids [17]. Aducanumab has been shown to selectively bind to parenchymal amyloid over vascular amyloid [18, 19]. The initial package insert of aducanumab generally states that the drug is indicated to treat AD even though the drug is only tested in early-onset AD patients. Hence, the U.S. FDA has recently approved an updated indication, which indicates that aducanumab should be initiated in patients with MCI due to AD or mild Alzheimer's dementia. After an initial titration period, aducanumab should be administered at a dose of 10 mg/kg, given as a monthly intravenous infusion. Aducanumab is absorbed and reaches a Cmax of 182.7 *μ*g/mL, with a Tmax of 3.0 hours, and an AUCinf of 31,400 h∗*μ*g/mL [20]. The drug achieves steady-state concentrations after 16 weeks of repeated dosing and a mean volume of distribution of 9.63 L. Aducanumab is eliminated after being broken down into smaller oligopeptides and amino acids [21]. The mean clearance and the terminal half-life for the drug are 0.0159 L/h and 24.8 days, respectively. Bodyweight, age, sex, and race seem to have no clinically significant impact on the exposure to the drug. Aducanumab may cause allergic reactions and amyloid-related imaging abnormalities (ARIA). ARIA events most commonly (80%) occur without any symptoms [12]. Even if symptoms appear, they include edema or microhemorrhages of the brain, which are transient adverse effects that may be reversed upon discontinuation of the drug [20].

## 4. Controversy on the Approval of Aducanumab

The approval of aducanumab by the U.S. FDA has resulted in a debate. Most patient advocates, AD patients, and their caregivers are pleased with the hope of having a new drug to treat AD [22], while some experts are against the approval of the drug due to multiple reasons [23, 24]. Firstly, the dug was approved based on surrogate endpoints and the modest impact on cognition [23, 25]. Furthermore, aducanumab is only tested in selected AD patients experiencing MCI or early-onset AD dementia, limiting its broader indications for all patients with AD. The lack of a confirmed causal relationship between *β*-amyloid plaques and cognitive decline may explain the lack of significant clinical impact [26]. However, it is important to note that there is no consensus on the definition of the minimum clinically significant difference (MCID) in Alzheimer's disease [27]. Inconsistent results have cast a doubt on the potential targeting of *β*-amyloid protein to improve cognitive and functional decline in AD patients [23, 28, 29]. Secondly, the experts argue that the risk associated with the drug may outweigh the benefit of the drug [14, 30]. According to Gleason et al., aducanumab dose-dependent transient amyloid-related imaging abnormalities (ARIA) may outweigh the slight improvement in cognitive decline attributed to the drug [31]. Thirdly, the drug is deemed to be not cost-effective with the initial annual acquisition cost of aducanumab which is $56,000 [26, 32, 33]. Recently, the manufacturer has declared an annual price cut ($28,200) for aducanumab to improve the uptake of this medication by patients with early-onset Alzheimer's. The cost associated with the drug is further increased by the need for screening and monitoring tests (PET imaging and periodic MRI) and drug administration costs. If the drug is ineffective, it may have a detrimental impact on human and economic resources [23]. Thirdly, approval of aducanumab may divert researchers away from potentially effective preventive and therapeutic measures [28]. Moreover, approval of an ineffective drug may destroy the people's trust in regulatory and licensing organizations [28]. A recent study by Anderson et al. assessed the representativeness of ENGAGE and EMERGE trial subjects by evaluating the proportion of Medicare patients with AD and MCI who would be excluded from these trials [34]. Based on this study, a large proportion of Medicare patients would not be eligible to receive aducanumab due to their comorbid conditions. This further raises a question regarding the broad indication of aducanumab for mild onset Alzheimer's disease. Regardless of the above concerns, the decision not to approve aducanumab might hinder investment by manufacturers involved in AD therapeutic research [12].

## 5. Studies That Evaluated the Efficacy or Safety of Aducanumab

A total of 11 studies were included in this review (Table 1); six of the reviewed studies are phase I clinical trials, one study is a phase II clinical trial, three studies are phase III clinical trials, and the remaining one study is a phase IV prospective observational study (Table 1). Most of the included studies were conducted in the U.S.A. (*n* = 7), 1 study was conducted in Japan, and the remaining three studies were conducted in more than one country (Table 1). The number of participants in these studies ranged from 21 [35] to 6000 [36].

The pharmacokinetics, safety, or tolerability of aducanumab was demonstrated using the following phase I trials: NCT01677572 [18], NCT02782975 [37], NCT01397539 [20], and NCT02434718 [35]. The latter trial investigated the drug's tolerability among Japanese patients [35]. Additional phase I open label trial assessing the bioavailability of aducanumab among healthy participants is currently underway (NCT04924140) and is expected to be completed on October 14, 2021 [38]. No results have been posted for this trial. In phase Ib PRIME trial [18], aducanumab was administered via monthly infusion to 165 patients with mild cognitive impairment and confirmed elevated *β*-amyloid plaques. This trial was conducted to check if aducanumab at different doses has an impact on clearing the *β*-amyloid deposition. The *β*-amyloid plaque was visualized using a PET scan; there was clear evidence of dose-dependent and treatment duration-dependent clearance of this plaque [39]. Patients in the higher dose group had a better clearance of *β*-amyloid. Amyloid-related imaging abnormalities (ARIA) such as ARIE (edema) and ARIH (hemorrhage) were observed among patients receiving higher doses of the drug, especially among patients who are APOE4 gene carriers. Moreover, a phase Ib clinical trial was conducted to assess the ability of PET scan in identifying *β*-amyloid plaques [40]. The study found that PET screening is a feasible and effective tool in identifying the *β*-amyloid plaques in AD patients [40].

The EVOLVE phase II trial (NCT03639987) [41] assessed the safety and impact of continuing aducanumab dosing in asymptomatic ARIA in participants with mild cognitive impairment (MCI) due to AD or with mild AD dementia. In addition, the study was aimed at characterizing ARIA from both the imaging and the clinical perspective and characterizing the safety, tolerability, pharmacokinetics (PK), and immunogenicity of aducanumab. The study was terminated based on the anticipated lack of impact of aducanumab in EMERGE [42] and ENGAGE [43] trials.

After the promising results from phase 1B (PRIME) trial [18], two identically designed phase III (ENGAGE [43] and EMERGE [42]) trials of 18-month duration were conducted to study if the clearance of *β*-amyloid plaques had an impact on delaying the progression of the cognitive impairment among patients with mild cognitive impairment (MCI) and early dementia. Both trials had patients with an average age of 70 years and included patients with APOE gene carriers and noncarriers. The trial used a clinical dementia rating scale to evaluate the impact of taking the drug in delaying the progression of the disease. The ENGAGE trial and EMERGE trials were terminated before completion due to lack of benefit based on data of the early 1748 patients in March 2019. The trials were not terminated due to safety concerns [44].

However, the reanalysis of larger data of 3285 patients showed there was a benefit with a higher dose in the EMERGE trial [45]. The patients in the EMERGE trial receiving high-dose aducanumab showed 22% improvement in adjusted mean clinical dementia rating scores [46]. Moreover, an 84% reduction of caregiver distress was also observed. Additionally, compared to the placebo group, 40% slowing of functional decline was noted through the ADCS-ADL scale and assessment by Neuropsychiatric Inventory (NPI) showed an 87% reduction in behavioral changes from baseline scores especially in the high-dose group of EMERGE. The ENGAGE trial on the contrary showed no dose-dependent benefit of drug therapy as compared to placebo [47]. The inconsistencies can be linked with fewer numbers of patients in the ENGAGE trial receiving higher doses of aducanumab. Based on this finding, the ENGAGE trial protocol was amended to allow patients with APOE4 gene carriers to receive higher doses. Subgroup analysis after protocol amendment showed similar results as the EMERGE trail [46]. However, the inconsistent results observed with aducanumab may be explained by the limited brain penetration and lack of selectivity for the soluble A*β*-oligomers, which are implicated as upstream drivers of neurodegeneration by multiple studies [48]. The most common adverse events reported within these two studies were ARIA, headache, diarrhea, and fall. ARIA-E was reported in 34% and 35.5% of patients who received high-dose aducanumab in EMERGE and ENGAGE, respectively.

A study by Salloway et al. reviewed the clinical and radiographic aspects of ARIA in both EMERGE and ENGAGE trials [49]. The study found that 425 of 1029 individuals (41.3%) developed ARIA during the placebo-controlled trial period, with 14 patients developing significant events (1.4%) [49]. ARIA-E was the most prevalent adverse event (362 of 1029 (35.2%)), with 263 initial incidents (72.7%) occurring during the first 8 doses of aducanumab use. The incidence of ARIA-E was noted to be greater in aducanumab-treated APOE4 carriers than in the noncarriers. However, the majority of ARIA-E-related occurrences (479 of 488 (98.2%)) were resolved radiographically. The most prevalent kind of ARIA-H was brain microhemorrhages (6.6% in the placebo and 19.1% in the 10 mg/kg group), followed by localized superficial siderosis (2.2% in the placebo and 14.7% in the 10 mg/kg group). The incidence of ARIA has caused 64 participants (6.2%) to discontinue the study. The discontinuation was more prevalent in APOE4 gene carriers than noncarriers [49].

Currently, three clinical programs are under development to generate postapproval real-world data about aducanumab. These include ICARE AD-US [36] study, ongoing phase IIIb redosing EMBARK study [50], and planned phase 4 postmarketing trial study that is currently under development.

## 6. Implications of Aducanumab Approval

The approval of the first disease-targeting antiamyloid *β*-drug is likely to inflate the hopes of patients with Alzheimer's disease. FDA's decision on the approval of aducanumab is of great importance to AD patients and their families, which can increase the pressure on clinicians to prescribe this drug. Hence, it is important to identify the right patients who are more likely to benefit from this drug. To maintain optimal effectiveness and safety of the drugs, patients who are eligible to take the drug via long-term monthly infusion need to be carefully selected. Before initiating the drug, a recent MRI and a PET scan to visualize the density of *β*-amyloid aggregates are needed [51]. Periodic safety MRI after the seventh and twelfth infusion and anytime when ARIA is suspected is required [18]. Hence, according to available data, patients with early-onset AD and MCI with clear *β*-amyloid plaques who are willing and able to undergo periodic follow-up with imaging studies such as PET and safety MRI and APOE4 genotype testing are good candidates for this drug.

The pressure on physicians to select patients who are most likely to benefit from the drug with available limited evidence [27] is anticipated to accelerate the development of less cumbersome and less costly serum-based tests to assess *β*-amyloid plaque. In addition, better collaboration with primary care centers is needed to screen and identify individuals at the early stage of the disease to offer disease-modifying intervention.

## 7. Future Directions in Treatment of Alzheimer's Disease

Future AD management is likely to focus on passive immunotherapy, vaccines, and early diagnosis based on neuroimaging, CSF, and plasma biomarkers.

AD CSF biomarkers have been the main diagnostic criteria for the disease. Recently, ultrasensitive immunoassays and novel mass spectrometry techniques have enabled the assessment of plasma biomarkers to monitor amyloid plaque formation (the A*β*42/40 or APP669-711/A*β*42 ratios) and neurodegeneration (tau and neurofilament light proteins) [52]. A similar study has also showed plasma P-tau217 has a potential to discriminate AD from other neurodegenerative diseases with comparable accuracy as key CSF- or PET-based measures. Another plasma biomarker, P-tau181, discriminated AD at MCI and dementia stages and was strongly associated with cognitive decline and gray matter loss [53]. Similarly, a study showed that U-p532D3A8+ can be used as additional plasma-based biomarker for AD [54]. These findings highlight the potential value of plasma biomarkers as a noninvasive and cost-effective screening, diagnostic, and monitoring tool. Before these assays can be used routinely, the assays need to be optimized and validated in several AD populations and its potential role in clinical care determined.

Several anti-amyloid beta and anti-tau therapies have been evaluated or are currently under evaluation. Anti-*β-*amyloid therapies act by reducing the pathologic *β*-amyloid oligomers or by inhibiting *β*-amyloid plaque formation or by increasing clearance of *β*-amyloid peptides. However, many of the trials on anti-*β*-amyloid therapies have failed to demonstrate clinical impact or pose safety concerns.

A few passive immunotherapies which act in a similar mode as aducanumab have been evaluated or are under evaluation. Currently, three clinical trials are assessing the efficacy and safety of lecanemab (NCT03887455, NCT04468659, and NCT01767311) in patients with AD. Another agent donanemab has demonstrated an improvement in composite cognition scores and ability to do activities of daily living (ADLs) in patients with early AD [55]. In addition, the trial that is assessing the safety and efficacy of gantenerumab among early AD patients is currently underway (NCT03443973, NCT03444870, and NCT01760005). Crenezumab's efficacy among preclinical AD patients is being tested in two clinical trials (NCT03977584, NCT01998841).

Agents that inhibit *β*-site amyloid precursor protein cleaving enzyme (BACE) (lanabecestat, elenbecestat, atabecestat, and verubecestat) have also been evaluated in individuals with mild to moderate AD and prodromal AD. The trials of BACE inhibitors have also failed to show clinical significance, and a trial was stopped due to safety concerns [56].

Anti-*β*-amyloid aggregating agent scyllo-inositol was ineffective among patients with mild to moderate AD while ALZ-801 is under investigation (NCT04770220). Currently, a small molecule, GAL-101, which can inhibit toxic *β*-amyloid oligomers, is under development. If proven effective, this molecule may provide a more convenient alternative oral therapy to patients with AD with less antibody-related adverse reactions such as allergies [57].

Failure of several agents that act on *β*-amyloid aggregates has forced researchers to shift the focus of potential to anti-tau therapies [58]. It is hypothesized that tau tangles are more correlated than *β*-amyloid aggregates with Alzheimer's disease pathogenesis [59]. Despite the hypothesis, anti-tau drugs that act through inhibition of tau formation, aggregation, and stabilizing microtubule have failed to demonstrate significant efficacy and some are associated with adverse effects [60–62]. However, it is important to note that patients who have *β*-amyloid aggregates are more likely to have tau tangles [63].

Vaccines against tau tangles have demonstrated modest clinical benefits [64]. This age-related diminished immune response in the elderly patients diagnosed with AD makes vaccines less effective. Therefore, vaccines for the elderly might require a potent adjuvant to enhance the immune response [65]. However, vaccines provide cheaper and affordable alternatives to drugs [65, 66].

An active vaccine (AADvac1) which acts against nonphosphorylated tau was evaluated in the phase I trial. The vaccine was found to be safe and well tolerated and triggered high levels of IgG antibody responses and significantly reduced neurofibrillary tangles. The vaccine slowed the degeneration of the brain by about 30%. It also significantly reduced 58% accumulation of neurofilament light chains in the blood [67]. However, the need for frequent booster doses is the main limitation of this vaccine. In addition, another active vaccine (ACI-35) that works on phosphorylated tau tangles is currently under investigation (NCT04445831). Additionally, the anti-amyloid E22W42-sensitized DC vaccine is also currently under development. The antigen in this vaccine will stimulate a highly specific T cell response, thereby destroying *β*-amyloid, while inhibiting harmful activities that may lead to autoimmunity. Along with slowing down the memory impairment of AD patients, it may help to strengthen the immune system of elderly patients [68].

In addition to active vaccines, the following passive immunization alternatives are under evaluation. These include agents such as RG7345, gosuranemab, tilavonemab, zagotenemab, semorinemab, BIIB076, JNJ-63733657, and bepranemab. The trial of RG7345 was ceased due to pharmacokinetic issues (NCT02281786). On the other hand, the phase II clinical trials in AD patients for gosuranemab (NCT03352557), for JNJ-63733657 (NCT04619420), and for semorinemab (NCT03828747) are still ongoing. Although trials of tilavonemab (ABBV-8E12) (NCT02880956, NCT03712787) were completed in 2021, no results are available. The phase II study of bepranemab (UCB 0107) involving AD patients (NCT04867616) is now recruiting.

Anti-tau compounds (epothilone, TPI287, and davunetide) which act by stabilizing tau microfilaments were also tested. The phase I trial of epothilone was discontinued. Similarly, the compound TPI287 was associated with an increased risk of hypersensitivity reaction in patients with AD [61]. On the other hand, davunetide failed to significantly improve the composite cognitive memory scores in patients with AD and this drug was associated with tauopathies [69]. Similarly, no significant benefits for patients with AD were observed after use of methylene blue [70] and curcumin (NCT01383161) which act by inhibiting tau aggregation. Moreover, trials of antitau drugs that inhibit glycogen synthase kinase (GSK 3) (lithium and tideglusib) in patients with mild AD also failed to show significant benefit [60, 62].

Since AD pathogenesis is multifactorial, drugs, which act on the combination of these targets, also need to be developed [9, 54]. In the future, there is a need for trials to demonstrate cognitive and functional benefits rather than focusing only on surrogate endpoints [28]. Neuroinflammation, metabolic disorder, infection, and genetic modifications may provide new targets for Alzheimer's disease management [26, 71]. Multiple interventions such as risk factors or lifestyle modification such as healthy nutrition, exercise, rest, social participation, and cognitive activity are anticipated to effectively halt the progression of the disease [72].

## 8. Conclusions

Aducanumab at a high dose has the potential to slow down the cognitive decline linked with Alzheimer's in patients with early-onset disease. However, aducanumab does not reverse memory loss. The approval of the drug has made patients with Alzheimer's disease hopeful but raised lots of doubts regarding its true benefit. The manufacturer is expected to verify the clinical benefit of aducanumab therapy to delay disease progression through an extended study. Aducanumab therapy will be limited by the need for prior authorization, intravenous administration, and uncertainties regarding benefit-risk ratio and cost-effectiveness. Despite its drawbacks, aducanumab is the first disease-modifying drug approved for AD. It is important to note that this drug only partially addresses the underlying pathology of AD. Hence, future interventions for AD require incorporating multiple strategies to effectively treat the disease.

## 9. Limitation

This narrative review did not include studies indexed in databases other than Google Scholar, Web of Science, and MEDLINE.

## Figures and Tables

**Table 1:** Studies that evaluated the efficacy or safety of aducanumab.

Trial name, reference, and NCT no.	Country	Design	Participants	Intervention	Results
PRIME (multiple dose study of aducanumab (BIIB037) (recombinant, fully human anti-A*β* IgG1 mAb) in participants with prodromal or mild Alzheimer's disease) [18] NCT01677572	32 sites in the U.S.A.	RCT phase 1b placebo-controlled multiple dose study	197 patients with prodromal or mild AD	Dose-escalation trial (4 aducanumab doses (1, 3, 6, and 10 mg/kg)) 40 patients from both groups discontinued treatment	165 patient analysis of PET scan showed dose- and duration-dependent reduction of amyloid plaque Terminated after futility analysis of ENGAGE and EMERGE trial on March 21, 2019
Absolute bioavailability of a single fixed subcutaneous dose of aducanumab in healthy participants [37] NCT02782975	2 sites in the U.S.A.	RCT phase 1 open label trial to assess absolute bioavailability	28 healthy volunteers	Single, fixed subcutaneous dose of aducanumab	No results are available Started on May 26, 2016, and completed on Jan 13, 2016
Single ascending dose study of BIIB037 in participants with Alzheimer's disease [20] NCT01397539	3 sites in the U.S.A.	RCT phase I placebo-controlled single ascending dose study	A total of 53 patients with probable AD with MMSE score of 14–26	Aducanumab (*n* = 39) and placebo (*n* = 14) Aducanumab was given at doses 0.3, 1, 3, 10, 20, 30, and 60 mg/kg	Aducanumab has adequate safety and tolerability profile and linear PK at doses≤30 mg/kg
Amyloid PET screening for enrichment of early-stage Alzheimer disease clinical trials: experience in a phase 1b clinical trial [40]	33 sites in the U.S.A.	RCT phase 1b, multicenter, placebo-controlled, multiple-dose study of aducanumab	278 patients with an evaluable PET scan	Ability of PET scan as a tool to identify amyloid plaque-positive patients	Interreader and intrareader agreements from visual readings were 98% and 100%, respectively. Amyloid PET imaging is an effective and feasible screening tool for enrollment of amyloid-positive patients with early stages of AD into clinical trials
PROPEL (single and multiple ascending dose study of aducanumab (BIIB037) in Japanese participants with Alzheimer's disease) [35] NCT02434718	7 sites in Japan	RCT phase I placebo-controlled, single and multiple ascending dose study	21 Japanese patients with mild to moderate AD To evaluate safety, tolerability, and PK	Single and multiple IV infusion of aducanumab Versus placebo	Started on June 24, 2015, and was completed on December 9, 2016 No results were posted
A study to assess absolute bioavailability of aducanumab in healthy volunteers [38] NCT04924140	2 sites in the U.S.A.	Phase I open label interventional trial to assess bioavailability	30 healthy participants	No results are available	Started on June 11, 2021 Expected to be completed on October 14, 2021
EVOLVE study (a study of aducanumab in participants with mild cognitive impairment due to Alzheimer's disease or with mild Alzheimer's disease dementia to evaluate the safety of continued dosing in participants with asymptomatic amyloid-related imaging abnormalities) [41] NCT03639987	22 sites in the U.S.A.	RCT phase II double-blind, controlled study of aducanumab	52 patients with mild MCI or mild AD dementia To evaluate safety of continuing aducanumab dosing in asymptomatic ARIA	Aducanumab	Terminated following futility analysis of ENGAGE and EMERGE trials
ENGAGE (221AD301 phase 3 study of aducanumab (BIIB037) in early Alzheimer's disease) [43] NCT02477800	181 sites from 14 countries (U.S.A., France, Australia, Spain, Austria, Canada, Denmark, U.K., Germany, Italy, Japan, Korea, Portugal, and Taiwan)	RCT phase III double-blind, placebo-controlled, parallel-group study	1647 patients with mild cognitive impairment or mild Alzheimer's dementia	Comparison of low-dose aducanumab and high-dose aducanumab and placebo	CDR sum boxes were not different Terminated due to anticipated lack of benefit
EMERGE (221AD302 phase 3 study of aducanumab (BIIB037) in early Alzheimer's disease) [42] NCT02484547	180 sites from 13 countries (Belgium, Italy, Canada, Finland, France, Sweden, Germany, Japan, Poland, Spain, Switzerland, Netherlands, and U.S.A.)	RCT phase III double-blind, placebo-controlled study	1638 patients with early Alzheimer's disease with confirmed amyloid pathology	Comparison of low- and high-dose aducanumab and placebo	High-dose aducanumab reduced clinical decline as measured by CDR-SB at 18 months and MMSE, ADAS-Cog 13, ADCS-ADL-MCI
EMBARK (a study to evaluate safety and tolerability of aducanumab in participants with Alzheimer's disease who had previously participated in the aducanumab studies 221AD103, 221AD301, 221AD302, and 221AD205) [50] NCT04241068	229 sites from 20 countries (Australia, Austria, Belgium, Canada, Denmark, Finland, France, Germany, Italy, Japan, Korea, Netherlands, Poland, Portugal, Spain, Sweden, Switzerland, Taiwan, U.K., and U.S.A.)	RCT phase IIIb with a 24-month treatment period	Planned enrollment of 2400 participants	It will evaluate the long-term safety and efficacy of aducanumab in participants with AD, who will be titrated to receive 10 mg/kg aducanumab by intravenous infusion every 4 weeks	AEs leading to treatment discontinuation or study withdrawal; amyloid-related imaging abnormality-edema (ARIA) or amyloid-related imaging abnormality-hemorrhage or superficial siderosis; and the number of participants with antiaducanumab antibodies
ADUHELM ICARE AD-US study (the first real-world observational phase 4 study in Alzheimer's disease at AAIC 2021) [36] NTC pending	Approximately 200 sites in the U.S.A.	Observational phase IV prospective, multicenter, noninterventional real-world study	Expected to enroll 6000 participants for 4 years, with at least 16% Latinx and Black/African American patients	The study is aimed at evaluating long-term changes in cognition, function, and neuropsychiatric status among patients treated with aducanumab 100 mg/mL solution for injection	The confirmatory phase IV trial is still under process of being designed and it is expected to monitor the participants for up to 5 years

Abbreviation: AD: Alzheimer's disease; ADAS-Cog 13: Alzheimer's disease assessment scale–cognitive subscale (13-item); ADCS-ADL-MCI: Alzheimer's disease cooperative study–activities of daily living inventory; ARIA: amyloid-related imaging abnormalities; CDR-SB: clinical dementia rating–sum of boxes; MCI: mild cognitive impairment; MMSE: mini-mental state examination; PET scan: positron emission tomography scan; RCT: randomized control trial; U.K.: United Kingdom; U.S.A.: United States of America.
